# Draft Genome Sequence of White Spot Syndrome Virus Isolated from Cultured *Litopenaeus vannamei* in Mexico

**DOI:** 10.1128/genomeA.01674-15

**Published:** 2016-03-10

**Authors:** Libia Zulema Rodriguez-Anaya, Jose Reyes Gonzalez-Galaviz, Ramón Casillas-Hernandez, Fernando Lares-Villa, Karel Estrada, Jose Cuauhtemoc Ibarra-Gamez, Alejandro Sanchez-Flores

**Affiliations:** a Departamento de Ciencias Agronómicas y Veterinarias, Instituto Tecnológico de Sonora, Ciudad Obregón, Sonora, Mexico; b Unidad de Secuenciación Masiva y Bioinformática, Instituto de Biotecnología, Universidad Nacional Autónoma de México, Cuernavaca, Morelos, Mexico

## Abstract

The first genome sequence of a Mexican white spot syndrome virus is presented here. White spot syndrome is a shrimp pandemic virus that has devastated production in Mexico for more than 10 years. The availability of this genome will greatly aid epidemiological studies worldwide, contributing to the molecular diagnostic and disease prevention in shrimp farming.

## GENOME ANNOUNCEMENT

In Mexico, shrimp farming is the most important aquaculture economic activity. However, its sustainability has been at risk in the last decade due to the low production yields triggered by viral outbreaks (1). Presently, white spot syndrome virus (WSSV) is the most devastating shrimp viral pathogen worldwide, causing mortality of up to 100% of farm production within 2 to 10 days (2). Classified under a new genus, *Whispovirus*, in the family *Nimaviridae* (3), WSSV is extremely virulent and has a wide host tissue tropism (4). Currently, there are four WSSV complete reference genomes deposited in the public databases, with differences in length: WSSV-Taiwan is 307,287 nucleotides (5), WSSV-China is 305,107 nucleotides (6), WSSV-Thailand is 292,967 nucleotides (7), and WSSV-Korea is 295,884 nucleotides (8). However, there is a lack of information related to WSSV functional genomics because of its taxonomical and sequence uniqueness. More than 90% of the predicted open reading frames (ORFs) have no significant similarity to any known proteins (4). Besides, there is evidence that demonstrates the existence of strain variability and differences in pathogenicity among geographical isolates of WSSV (9–13). Therefore, we sequenced, assembled, and analyzed the whole genome of a WSSV Mexican strain (WSSV-MX08), which is the first isolate from Mexican shrimp farms. WSSV-infected shrimp were collected from shrimp ponds in Sonora State. Total DNA was extracted with the GeneJET genomic DNA purification kit, and a library was prepared to be sequenced in the Ion Torrent PGM platform using a 316 Chip, according to the vendor’s protocol. We obtained a total of 1,435,498 reads, with a maximum read length of 400 bases, which were assembled in 7,228 contigs. Using the ABACAS (14) and Mauve (15) programs, we aligned the contigs to the four reference genomes available to separate those belonging to WSSV-MX08 from the shrimp genome contigs. Using the alignment information from ABACAS, we reconstructed the whole virus genome by ordering and orienting the contigs, using the WSSV-Thailand reference genome sequence as the template. The G+C content was 41%, which is the same as that of the other WSSV genomes (8), and the average nucleotide identity (ANI) (16) between WSSV-MX08 and the other genomes is between 99.5% and 99.64%, with the WSSV-Korea genome being the most distant. For coding sequence comparison, we used the Artemis genome browser version 16.0.0 and found a lack of ORFs 122 and 123 in WSSV_MX08, which are also missing in WSSV-Korea (8). The nucleocapsid protein VP35 is also absent, as observed in the WSSV-Thailand (17) and WSSV-Korea (8) strains, suggesting that this protein is not essential for viral replication. One of the biggest difference reported among the isolates is a genetic variation in ORFs 14 and 15 (17), as a 575-bp deletion in this region was observed in WSSV-MX08. In order to understand the evolution of WSSV, further studies are needed to characterize the genomic variations in a protein context and to and associate them with other variables, such as the geographical distribution, virulence phenotypes, host-virus interactions, and quasispecies modeling in populations.

### Nucleotide sequence accession number.

This whole-genome shotgun project has been deposited at DDBJ/EMBL/GenBank under the accession no. KU216744.
